# Sex differences in circulating platelet‐derived CD41+ extracellular vesicles in healthy adults

**DOI:** 10.14814/phy2.70932

**Published:** 2026-07-03

**Authors:** Garett Jackson, Hashim Islam, Alexandre Abilio de Souza Teixeira, Christopher DeSouza, Isaac T. S. Li, Jonathan Little

**Affiliations:** ^1^ School of Health and Exercise Science, Faculty of Health and Social Development The University of British Columbia Kelowna British Columbia Canada; ^2^ Immunometabolism Research Group, Institute of Biomedical Sciences University of Sao Paulo (USP) Sao Paulo Brazil; ^3^ Integrative Vascular Biology Laboratory, Department of Integrative Physiology University of Colorado Boulder Boulder Colorado USA; ^4^ Department of Chemistry University of British Columbia Kelowna British Columbia Canada

**Keywords:** extracellular vesicles, microRNA (miR‐126, miR‐146a, miR‐155, miR‐181b), microvesicles, monocyte‐derived EVs (CD14+), platelet‐derived EVs (CD41+), sex differences

## Abstract

Extracellular vesicles (EVs) facilitate intercellular communication and reflect the physiological state of their parent cells. Microvesicles (MVs; ~200–900 nm) are released during cellular activation or stress and may provide insight into sex‐related physiological differences. This study aimed to characterize circulating MV concentration, cellular origin, and MV microRNA cargo in healthy young males and females. Fasted sodium citrate plasma samples from healthy male (*n* = 16, age: 29 ± 6 years, BMI: 24.4 ± 2.4 kg/m^2^) and female (n = 16, age: 27 ± 5 years, BMI: 22.7 ± 1.9 kg/m^2^) participants were analyzed using size exclusion chromatography, tunable resistive pulse sensing, nano‐flow cytometry, and RT‐qPCR. MV concentration, antibody‐defined MV subpopulations, and MV‐miRNA cargo were assessed using linear models adjusted for age and BMI. Females exhibited higher concentrations of circulating platelet‐derived CD41+ MVs (*p* = 0.03, *d* = 0.923), with a tendency for elevated monocyte‐derived CD14+ MVs (*p* = 0.08, *d* = 0.546). No differences were observed for total MV concentration, remaining antibody‐defined MV subpopulations, or MV‐miRNA cargo expression. These findings demonstrate that healthy females exhibit elevated platelet‐ and monocyte‐derived MV concentrations, while overall MV concentration and MV miRNA cargo are similar between sexes.

## 1 INTRODUCTION

Biological sex is a fundamental regulator of vascular and immune physiology, shaping endothelial function, platelet reactivity, inflammatory signaling, and nitric oxide (NO) bioavailability; processes that collectively influence cardiometabolic risk and immune responsiveness (Klein & Flanagan, 2016; Ranucci et al., 2019; Stanhewicz et al., 2018). These sex‐related differences are partly mediated by sex hormone signaling and downstream regulation of pathways involved in vascular tone and inflammatory activation, including endothelium‐dependent vasodilation and nuclear factor kappa B (NF‐ĸB) linked transcriptional programs (Hoffmann et al., 2023; Orshal & Khalil, 2004; Stanhewicz et al., 2018). Sex hormones may also influence platelet activation and platelet‐endothelial interactions, suggesting a plausible mechanistic basis for sex‐specific variation in circulating platelet‐derived extracellular vesicles (EVs) under resting conditions (Hadley et al., 2022; Khetawat et al., 2000; Nakano et al., 1998). Because EVs are released during cellular activation, stress, and membrane remodeling, circulating EV profiles may provide an integrated readout of sex‐specific vascular and immune states (Hooten et al., 2022; Jansen et al., 2013; Shah et al., 2018). However, the extent to which EV concentration and cell‐origin subpopulations differ between healthy males and females at baseline remains incompletely defined, in part due to heterogeneity in EV isolation, sizing thresholds, and phenotyping strategies across studies (Gustafson et al., 2015; Hooten et al., 2022; Welsh et al., 2024).

EVs are membrane‐bound particles released from cells during activation, stress, or apoptosis and serve as mediators of intercellular communication (Shah et al., 2018; Valadi et al., 2007). EVs encompass multiple subtypes that differ in biogenesis and size, historically referred to as exosomes, ectosomes, and microvesicles (MVs) (Welsh et al., 2024). Current consensus guidelines recommend use of the generic term “EVs”, with size‐based categorization into small (<200 nm) and large (>200 nm) vesicles (Welsh et al., 2024). In the present study, we focus on EVs approximately 200–900 nm in diameter and refer to this population as MVs, consistent with prior vascular physiology literature (Brewster et al., 2020; Garcia et al., 2025; Stockelman et al., 2020).

Several studies have examined sex‐based differences in circulating EVs; however, findings remain inconsistent and are often secondary to broader study aims (Hooten et al., 2022; Shill et al., 2018). In healthy cohorts, endothelial‐ and platelet‐derived EV subpopulations have been reported to differ between males and females, with some studies observing higher concentrations in males (Bammert et al., 2017; Shill et al., 2018; Stockelman et al., 2020), whereas others report elevated leukocyte‐derived EVs in females (Hooten et al., 2022). Importantly, many investigations have adjusted for sex without directly analyzing baseline differences or including mixed disease populations (Hijmans et al., 2019; Landers‐Ramos et al., 2020). In addition, substantial heterogeneity exists across studies with respect to EV isolation approaches, sizing thresholds, flow cytometry gating strategies, and marker panels (Welsh et al., 2024). As a consequence, whether biological sex independently influences circulating MV concentration and phenotype under standardized analytical conditions remains unclear.

EVs transport micro ribonucleic acids (miRNAs), providing a protected, cell‐informative cargo that can modulate gene expression in recipient cells and may reflect upstream vascular and immune activation states (Valadi et al., 2007). Numerous miRNAs have been identified in EVs, where they may reflect the molecular state of the parent cell and, upon transfer, influence gene regulation in recipient cells (Valadi et al., 2007). Among candidate EV‐miRNAs relevant to cardiometabolic and inflammatory physiology, miR‐126 is frequently linked to endothelial integrity and angiogenic signaling, making it a plausible indicator of endothelial stress or remodeling (Zhang et al., 2013). miR‐146 and miR‐155 are widely implicated in immune regulation and inflammatory signaling networks, including pathways downstream of Toll‐like receptor activation and NF‐κB transcriptional programs, and may therefore help contextualize EV signatures associated with systemic inflammation (O'neill et al., 2011; Prattichizzo et al., 2021). miR‐21 is commonly associated with cellular stress responses and vascular remodeling processes, while miR‐92 has been linked to endothelial activation phenotypes and regulation of vascular homeostasis (Bonauer et al., 2009; Nemecz et al., 2023). Finally, miR‐181b has been implicated in immune cell differentiation and inflammatory responsiveness and may provide complementary insight into sex‐related differences in immune‐vascular crosstalk (Dehghani et al., 2020; Sun et al., 2012). Collectively, profiling EV miRNA cargo alongside EV concentration and cell‐origin phenotyping offers a mechanistically grounded approach to interpret circulating EV patterns as integrative biomarkers of vascular and immune physiology.

To address the limited and inconsistent evidence regarding sex‐related differences in circulating MV profiles in healthy adults, the purpose of this study was to compare plasma MVs in the 200–900 nm size range between young, healthy females and males. Using a combination of size exclusion chromatography (SEC), tunable resistive pulse sensing (TRPS), nano‐flow cytometry, and quantitative polymerase chain reaction (qPCR), we quantified MV concentration, characterized antibody‐defined MV subpopulations, and assessed selected MV miRNA cargo. We tested the hypothesis that plasma MV profiles and characteristics would differ between females and males under standardized analytical conditions.

## 2 METHODS

### 2.1 Participants

In this investigation, 16 females and 16 males (*N* = 32; self‐reported biological sex assigned at birth) volunteered to take part in the study. To be included, participants (1) were between 18 and 40 years of age; (2) had a body mass index (BMI; kg/m^2^) between 18.5 and 30; (3) were recreationally active such that they completed ≤3 structured exercise sessions per week; (4) were non‐cigarette smokers for a minimum of 6 months prior to taking part in the study; (5) had no prior history of cardiometabolic (e.g., diabetes, heart disease) or inflammatory/autoimmune disease (e.g., Crohn's disease). Participants could not take part in the trial if they (1) competitively trained for an endurance sport; (2) had reported being sick within the previous 3 weeks; (3) were using anti‐inflammatory, immunomodulatory, or glucose‐lowering medications; (4) followed an extreme diet (e.g., ketogenic diet, time restricted feeding); were unable to travel to/from the lab; (5) unable to follow outlined pre‐experimental physical and dietary controls; (6) pregnant or planning to become pregnant during the study. Participants completed a 3‐day dietary food log as well as the International Physical Activity Questionnaire (IPAQ). Females completed a menstrual questionnaire, with blood samples taken during the early follicular phase of their menstrual cycle. Additionally, females provided information regarding the use of oral contraceptives. All participants provided written informed consent prior to taking part in the study. The study was conducted in accordance with the Declaration of Helsinki and was approved by the University of British Columbia Clinical Research Ethics Board (H20‐00240).

### 2.2 Laboratory visit and blood sampling

Participants arrived at the laboratory between 8:00 and 9:00 am following an ≥8‐h overnight fast. Participants were directed to abstain from exercise for at least 24 h prior to their laboratory visit. Following collection of participant height, weight, and blood pressure, a venous blood sample was collected using a 21‐gauge butterfly needle first into EDTA‐coated vacutainers, and then into sodium citrate (0.109 M, 3.2%) coated vacutainers (Becton Dickinson, USA) in which the applied tourniquet duration did not exceed 30 s. Samples were inverted 6–8 times and transported upright at room temperature (21°C) to the adjacent wet laboratory for processing. All blood samples were centrifuged immediately following blood collection.

### 2.3 Blood and plasma processing

Fresh sodium citrate blood samples were centrifuged (Eppendorf 5430R) at 2500×*g* for 15 min at 4°C to obtain platelet poor plasma. The upper 50% of plasma volume from two collection tubes was first combined, mixed, and then transferred to two 450 μL aliquots and stored at −80°C for batch analysis. Plasma was thawed in the refrigerator prior to analysis. Samples were spun at 13,000×*g* for 2 min (Eppendorf 5430R) at room temperature to remove any remaining platelets and debris prior to introduction of antibodies. 200 μL of the uppermost plasma was transferred to a new sterile microtube, which was gently mixed by pipetting and served as the source for analysis. 50 μL of plasma was transferred to 3 separate sterile microtubes. Prior to staining, the individual antibodies were spun at 10,000×*g* for 2 min (Eppendorf 5430R) to remove aggregates and dissociated fluorophores. The first microtube, P1 (Panel 1), was left unstained and served as the control panel. Panel 2 received 2 μL each of CD3 PE (130‐114‐519, Miltenyi Biotec), CD14 PerCP‐Vio700 (130‐110‐523), CD16 PE‐Vio615 (130‐119‐995), CD31 FITC (130‐110‐668), CD41 APC (130‐123‐301), and 10 μL of CD62E PE (130‐104‐643). These antibodies were selected from the Miltenyi REAfinity antibody portfolio, which is designed to reduce non‐specific Fc receptor‐mediated binding. Based on prior pilot optimization, FcR block did not materially alter staining patterns for these antibodies and was therefore not used for Panel 2. Panel 3 received 10 μL of Miltenyi FcR block (130‐059‐901) for 15 min prior to staining, followed by 10 μL of Annexin V FITC (130‐093‐060) and APOB100 PE (ITM0036‐50u‐594, G‐Biosciences). FcR block was used in Panel 3 because APOB100 was not part of the Miltenyi REAfinity antibody panel. Following incubation, each panel was brought to 500 μL using sterile 0.1 μm filtered 1x PBS (Cytiva, Utah, USA) and processed through an IZON qEV70 column. For MVs of interest in the 200–900 nm diameter range, silica nanospheres, which maintain a similar refractive index compared to EVs, were used as a reference material (Varga et al., 2018).

### 2.4 EV isolation by size exclusion chromatography (SEC)

Following plasma MV labeling, the 500 μL samples were processed using the IZON qEV Original 70 nm columns with the IZON automatic fraction collector (AFC). Early pilot/optimization work informed our choice of staining prior to SEC processing due to substantial reductions in background noise as the column reduced the presence of small proteins and debris, enabling them to be excluded for analysis. Each participant was processed using their own column to ensure consistency between each individual's specific panels. Prior to adding sample, each column was flushed with 15 mL of 0.1 μm PBS. Once the column was flushed, P1 (500 μL) was added to the top of the column, with 6 mL of 0.1 μm filtered 1× PBS added once the sample had passed through the upper frit. The sample was collected into 6 X 500 μL fractions, with the initial 2.7 mL discarded. Pilot work demonstrated that fractions 1–5 contained EVs, with fraction 6 containing a mixture of small EVs, proteins, debris, and unbound antibody. Fraction 6 was discarded and not analyzed on the flow cytometer or TRPS. Prior to P2 and P3 processing, the column was cleaned with 15 mL of 0.5 M NaOH and flushed with 25 mL of 0.1 μm 1× PBS. Following fraction collection, 100 μL of each fraction was added to a master mix and diluted 2‐fold to achieve a final volume of 1 mL (120× final dilution from plasma). The samples were promptly analyzed on the CytoFlex S flow cytometer (details below).

### 2.5 Flow cytometry

To analyze MVs, we used the CytoFLEX S (Beckman Coulter, USA) with a 405 nm violet side scatter configuration. At the start of each day, the flow cytometer was thoroughly cleaned using the daily clean option with prolonged run times (15 min Flow Clean, 15 min deionized [DI] water). Following the prolonged clean and preparation, the system was calibrated using the CytoFLEX Daily QC Fluorospheres (Cat No. B53230; Beckman Coulter, USA) as per manufacturer specifications. Experiments were all initiated using the same instrument settings and the system was further cleaned with 1% Contrad (Beckman Coulter, USA) in DI water for 5 min at 60 μL/min. Following this cleaning step, the system was flushed with DI water at the following rates and times: 240 μL/min for 5 min, 60 μL/min for 5 min, 30 μL/min for 5 min, and 10 μL/min for 15 min. The flow cytometer was ready for analysis once <400 events/s at 60 μL/min were observed for at least 3 min following this cleaning process. Between samples, 1% Contrad was run for 1 min at 60 μL/min, followed by 0.1 μm filtered water (HyClone) run for 2 min at 240 μL/min and 2 min at 60 μL/min. The next sample was only run once <400 events/s had been maintained at 60 μL/min for 1 min. There was no deviation in any system parameter (e.g. gain, gating, sample uptake speed) during the analysis of any of the samples. Antibody‐defined MV subpopulations were operationally defined as follows: CD3+ (T cell–derived), CD14+/CD16− (classical monocyte–derived), CD16+/CD14− (neutrophil‐derived), CD31+/CD41− (endothelial‐derived), CD41+/CD31− (platelet‐derived), CD31+/CD62E+ (activated endothelial–derived), and Annexin A5+ (phosphatidylserine‐exposing MVs). Samples were analyzed as either positive or negative for a specific marker determined by fluorescence minus one (FMO) controls following calibration and compensation. Events were collected between 200 (cat. DNG‐B006) and 900 nm (cat. DNG‐B041) as determined using CD Bioparticle (USA) silica beads possessing a similar refractive index to human EVs. Representative flow cytometry plots are provided in Figure S1.

### 2.6 Tunable resistive pulse sensing

A single aliquot was processed using the IZON Exoid (Christchurch, NZ). An NP300 nanopore was prepared using the automated nanopore setup procedure provided within IZON's Control Suite. All reagents were acquired through IZON and prepared specifically according to the manufacturer's directions. Once the nanopore was set up and ready for biological samples, 35 μL of sample processed through the SEC column was loaded into the upper fluid cell and the combined fractions 1–5 were analyzed. The calibration step was completed between every participant using the same prepared CPC400 beads diluted to 1 × 10^9^ particles/mL. The mean, mode and max diameters are provided as mean ± SD. CPC200 beads also diluted to 1 × 10^9^ particles/mL were used as a means of determining the lower end of sizing to ensure excessive events below 200 nm and above 900 nm were not included in the analysis.

### 2.7 miRNA extraction

To isolate MVs for miRNA extraction, plasma samples were processed using SEC to isolate EVs and reduce contamination from soluble proteins and lipoproteins, which are known to confound plasma‐derived EV analyses. Following SEC, samples were centrifuged at 25,000×*g* for 60 min at room temperature using a fixed‐angle rotor (Eppendorf 5430R) to enrich the desired MV population. This additional centrifugation step enabled concentration of SEC‐isolated MVs to increase miRNA yield for downstream qPCR analyses. Plasma MV miRNA was isolated using the miRNeasy Mini Kit 50 (Cat. No. 217004; Qiagen GmbH, Germany). Prior to any sample handling, the biosafety cabinet was sprayed with 70% ethanol and left to dry for 15 min. After drying, the area was sprayed thoroughly with RNase Erase (Cat. No. 112440204; MP Biomedicals, OH, USA). A 500 μL aliquot of undiluted sodium citrate plasma from the same master pool used for flow cytometry and TRPS was passed through an IZON qEV70 column into 6 fractions. Fractions 1–5 were pooled and combined into two sterile, nuclease‐free microtubes (~1250 μL each) and centrifuged at 25,000×*g* for 60 min at room temperature. All but ~100 μL of volume was removed from each tube, vortexed for 1 min on high, and combined to produce ~200 μL of EV‐rich PBS. A total of 700 μL of QIAzol Lysis Reagent (Cat. No. 79306; Qiagen GmbH, Germany), supplied with the miRNeasy kit, was added to the sample and vortexed on high for 2 min to lyse EVs, then incubated for 5 min at room temperature. Next, 140 μL of chloroform was added to the mixture, vortexed for 15 s on medium, and incubated for 3 min at room temperature. The sample was then centrifuged at 12,000×*g* at 4°C for 15 min. The upper aqueous phase was removed and transferred to a nuclease‐free microtube where 1.5 volumes of 100% ethanol were added. The remaining steps were completed as per protocol, and isolated miRNA was stored at −80°C prior to batch analyses.

### 2.8 RT‐qPCR

cDNA was diluted 1:30 in nuclease free water prior to analysis as per manufacturer instructions. qPCR was conducted using the miRCURY LNA SYBR Green PCR Kit (Cat. 339346, Qiagen, USA). Primers designed by Qiagen consisted of cel‐miR‐39‐3p (YP00203952), miR‐103a‐3p (YP00204063), miR‐126‐3p (YP00204227), miR‐146a‐5p (YP00204688), miR‐155‐5p (YP02119311), and miR‐181b‐5p (YP00204530). Cel‐miR‐39‐3p was chosen as the exogenous normalization miRNA that was added directly to the QIAzol prior to initiating the extraction protocol. Control samples were included that had primers but no template, and one with a template with no primers. The specific qPCR cycling conditions were as follows: initial heat activation for 2 min at 95°C, denaturation for 10 s at 95°C, and annealing and extension for 60 s at 56°C which was set to 40 cycles. A melting curve analysis concluded the run to determine reaction specificity. miRNA expression levels were determined by Cq values measured in duplicate.

### 2.9 Statistical analysis

Data were statistically analyzed using R Studio (version 4.2.2) using linear models with BMI and age included as covariates (lm(DV ~ sex + BMI + age, data = data)). Normality was assessed using histograms and Q–Q plots. Outliers were identified and removed using a 3× standard deviation (SD) criterion applied to the analysis variable prior to statistical modeling in R. After normality testing, if data were abnormally distributed as assessed by the Shapiro–Wilk test and visual inspection of residual and Q‐Q plots, they were log transformed. Log transformations were applied to MV concentration, CD3, CD14+/CD16−, CD16+/CD14−, CD31+/CD41−, and Annexin A5 outcomes, while CD45, CD41+/CD31−, and CD62E were analyzed on the original scale based on distribution characteristics. MV miRNA expression (miR‐126‐3p, miR‐146a‐5p, miR‐155‐5p, and miR‐181b‐5p) was normalized to the exogenous spike‐in control (cel‐miR‐39) using the ΔCq method (target—cel‐miR‐39), and relative expression was calculated using 2^‐ΔCq prior to analysis. Cohen's d was calculated using the “effectsize” package and is reported for all measures with the following interpretations: <0.2 = negligible, 0.2 = small, 0.5 = moderate, ≥0.8 = large. Effect estimates with 95% confidence intervals (CI95) of the effect estimate difference between females and males are provided for all measures along with alpha (α), which was set a priori to *p* < 0.05. As a secondary analysis of a previous study examining sex differences in immune function (Islam et al., 2022), a sample size calculation was not performed a priori for the present outcomes and adjustments for multiple comparisons were not applied. A sample size of *n* = 16 per group provided 80% power to detect a large effect size (Cohen's d = 1.02) at an alpha of 0.05. Descriptive statistics are presented in Table 1. Log‐transformed variables were used for statistical testing, while untransformed values are presented in figures for visualization. A.

**TABLE 1:** Participant characteristics of male and female participants.

	Male (*n* = 16)	Female (*n* = 16)	Total (*N* = 32)	*p*‐value
Age (years)	29 ± 6	27 ± 5	28 ± 6	0.256
Height (cm)	**181.1 ± 6.5**	**166.8 ± 5.6**	**173.9 ± 9.4**	**<0.001** *
Body Mass Index (kg/m^2^)	**24.4 ± 2.4**	**22.7 ± 1.9**	**23.8 ± 2.7**	**0.024** *
Systolic blood pressure (mmHg)	118 ± 7	116 ± 7	117 ± 7	0.592
Diastolic blood pressure (mmHg)	70 ± 8	74 ± 6	72 ± 7	0.105

*Note*: Values are presented as mean ± SD. Between‐sex comparisons were performed using independent samples *t*‐tests. Statistical significance was set at *p* ≤ 0.05. Significant values are bolded.

Abbreviations: cm, centimeters; kg/m^2^, kilograms per square meter; mmHg, millimeters of mercury.

* *p* < 0.05.

## 3 RESULTS

Participant characteristics are summarized in Table 1. Males were taller than females, and females had a lower BMI. No sex differences were observed for age, systolic blood pressure, or diastolic blood pressure.

### 3.1 MV size and concentration

**FIGURE 1:**
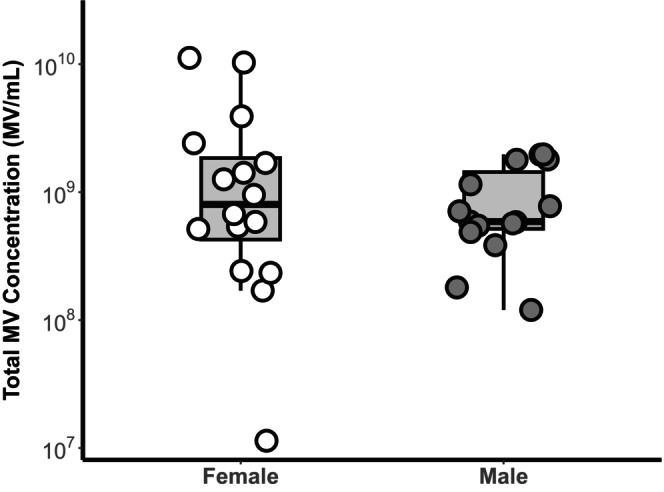
TRPS‐derived microvesicle (MV) concentration in males and females. Clear circles represent females and dark filled circles represent males. Statistical comparisons between sexes were performed using linear models adjusted for age and BMI. Data are presented as untransformed values for visualization following outlier removal using a 3*SD criterion. Statistical analyses were performed on log‐transformed data where appropriate.

A linear model adjusted for age and BMI revealed no significant difference in plasma MV concentration between males and females (Figure 1).

### 3.2 Antibody defined MV subpopulations

**FIGURE 2:**
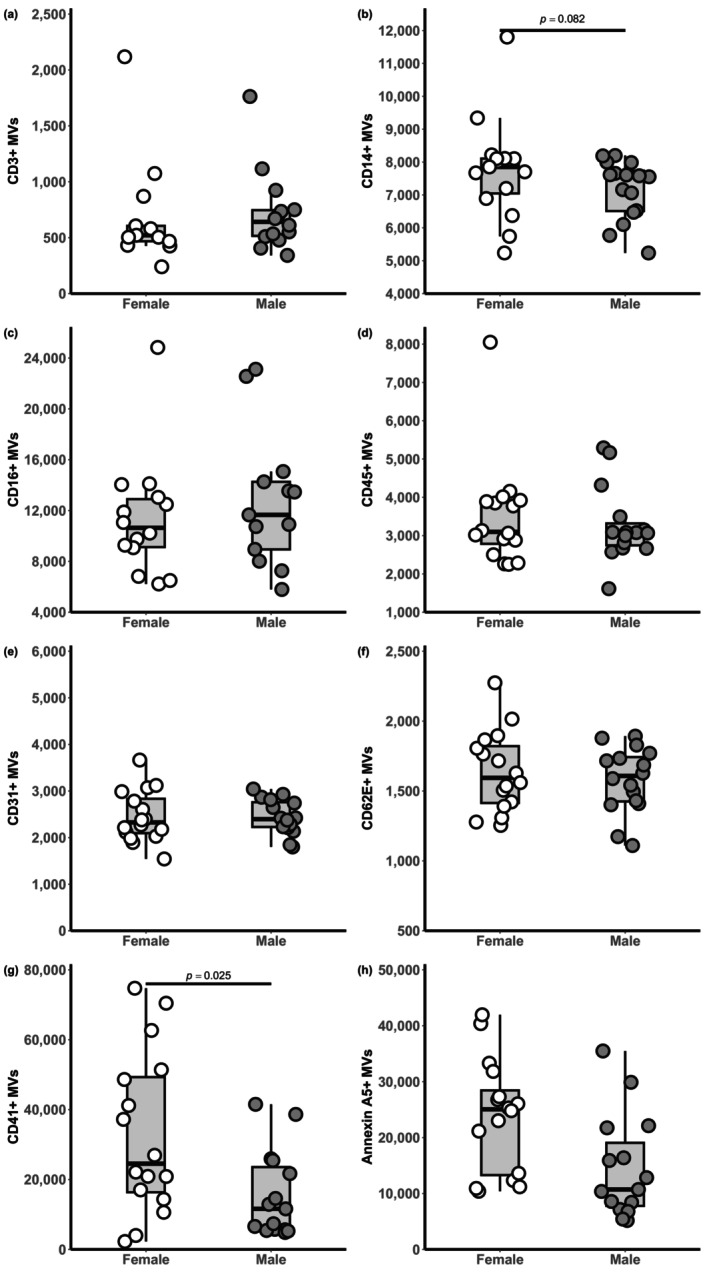
Nano flow cytometry measures of MV phenotype in males and females. (a) CD3+ lymphocyte‐derived MVs. (b) CD14+ monocyte‐derived MVs. (c) CD16+ neutrophil‐derived MVs. (d) CD45+ leukocyte‐derived MVs. (e) CD31+ endothelial cell‐derived MVs. (f) CD62E+ endothelial activation‐derived MVs. (g) CD41+ platelet‐derived MVs. (h) MVs expressing Annexin A5. Individual data points are presented as MV counts/μL. Clear circles represent females and dark filled circles represent males. Statistical comparisons between sexes were performed using linear models adjusted for age and BMI. Data are presented as untransformed values for visualization following outlier removal using a 3*SD criterion. Statistical analyses were performed on log‐transformed data where appropriate.

Antibody‐defined MV subpopulations are presented in Figure 2. CD41+/CD31‐ platelet‐derived MV concentration was significantly higher in females compared with males. There was a tendency for elevated circulating plasma CD14+/CD16− monocyte‐derived MVs in females, though this was not statistically significant. In contrast, no significant sex differences were observed for CD3+, CD16+/CD14−, CD31+/CD41‐, CD45+, CD31+/CD62E+, or Annexin A5+ MV subpopulations.

### 3.3 miRNA

**FIGURE 3:**
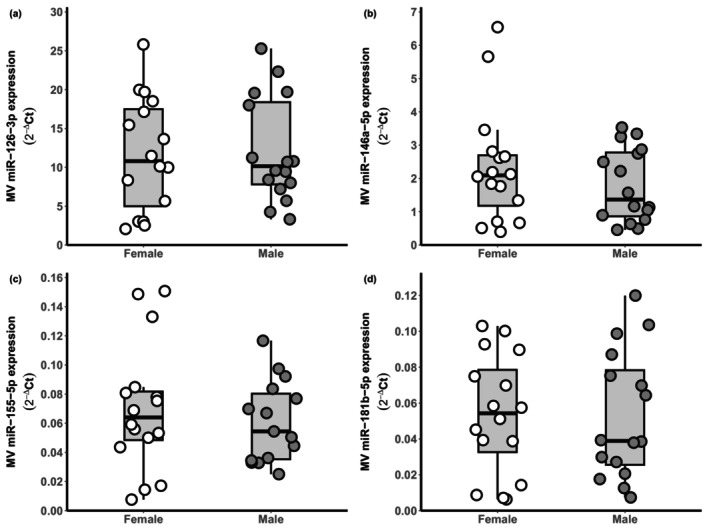
MV cargo containing miR‐126‐3p (a), miR‐146a‐5p (b), miR‐155‐5p (c), and miR‐181b‐5p (d) determined using RT‐qPCR performed on isolated MVs following size exclusion chromatography (SEC) and high‐speed centrifugal concentration. Clear circles represent females and dark filled circles represent males. Statistical comparisons between sexes were performed using linear models adjusted for age and BMI. Data are presented as untransformed values for visualization following outlier removal using a 3*SD criterion. Statistical analyses were performed on log‐transformed data where appropriate.

No significant sex differences were observed for miR‐126‐3p, miR‐146a‐5p, miR‐155‐5p, or miR‐181b‐5p (Figure 3). Effect estimates, confidence intervals, and effect sizes for all outcomes are presented in Table 2.

**TABLE 2:** Sex differences in MV phenotypes and MV microRNA cargo expression.

MV measure	Effect estimate (95%CI)	Cohen's d	*p*‐value
CD3+	0.03 (−0.48 to 0.53)	0.024	0.920
CD14+/CD16−	0.13 (−0.02 to 0.28)	0.546	0.082
CD16+/CD14−	−0.19 (−0.62 to 0.23)	0.469	0.354
CD31+/CD41−	0.001 (−0.16 to 0.16)	0.036	0.992
*CD41+/CD31*−	**18559.61 (2508.43 to 34610.77)**	**0.923**	**0.025** *
CD45+	418.94 (−581.09 to 1418.99)	0.188	0.398
CD31+/CD62E+	25.88 (−190.58 to 242.35)	0.223	0.808
Annexin A5+	0.25 (−0.16 to 0.66)	0.810	0.223
MV Concentration (count/mL)	0.12 (−1.02 to 1.25)	0.129	0.836
miR‐126‐3p	1.017 (−4.68 to 6.71)	0.062	0.717
miR‐146a‐5p	0.116 (−0.93 to 1.16)	0.214	0.821
miR‐155‐5p	0.011 (−0.02 to 0.04)	0.247	0.470
miR‐181b‐5p	0.002 (−0.03 to 0.03)	0.012	0.862

*Note*: Values represent model‐estimated differences between females and males with 95% confidence intervals derived from linear models adjusted for age and BMI. Effect sizes are reported as absolute values (Cohen's d) to reflect magnitude of difference and interpreted as negligible (<0.2), small (0.2–0.49), medium (0.5–0.79), or large (≥0.8). Statistical significance was set at *p* ≤ 0.05. Significant values are bolded.

Abbreviations: microRNA, micro ribonucleic acid; mL, milliliter; MV, Microvesicle.

* *p* < 0.05.

## 4 DISCUSSION

This study characterized circulating MVs within the 200–900 nm size range in young, healthy females and males under standardized isolation and analytical conditions. The primary finding was a statistically higher concentration of CD41+/CD31‐ platelet‐derived MVs with an observed tendency for increased CD14+/CD16− MVs in females compared with males although this failed to reach statistical significance. In contrast, total MV concentration, other antibody‐defined MV subpopulations, and MV expression of miR‐126‐3p, miR‐146a‐5p, miR‐155‐5p, and miR‐181b‐5p did not differ between sexes. These findings suggest that, in a healthy young cohort, sex‐related differences in circulating MV profiles are subtle and appear limited to higher plasma platelet‐derived MVs in females.

Platelets are a principal source of circulating MVs and release vesicles in response to cellular activation, shear stress, inflammatory signaling, and membrane remodeling (Boulanger et al., 2017; Brewster et al., 2020; Hunter et al., 2008; Koganti et al., 2017). CD41 (integrin αIIb) is a canonical platelet surface marker and reflects vesicles derived from platelet membranes, while the absence of CD31 reduces potential endothelial overlap within this phenotype of interest (Gustafson et al., 2015; Holcar et al., 2025; Toth et al., 2007). The elevated concentration of CD41+/CD31‐ MVs observed in females may therefore reflect sex‐related differences in platelet activation state, platelet‐endothelial interactions, or baseline platelet reactivity (Brewster et al., 2020; Gustafson et al., 2015; Toth et al., 2007). Prior investigations examining sex‐related differences in circulating EVs (and “microparticles”) have yielded inconsistent findings, with some studies reporting higher endothelial‐ or platelet‐derived populations in males and others observing elevations in specific MV subtypes in females, in part due to differences in cohort characteristics and analytical approaches (Bammert et al., 2017; Shill et al., 2018; Welsh et al., 2024). Indeed, many studies include mixed disease populations, apply variable sizing thresholds, or use limited marker panels, and sex is frequently adjusted for rather than directly evaluated at baseline (Holcar et al., 2025; Hooten et al., 2022). In the present study, standardized isolation and phenotyping with a comprehensive marker and miRNA panel provides a controlled assessment of baseline sex differences, supporting the interpretation that sex‐related variation in circulating MV profiles in healthy young adults is selective rather than systemic and appears predominantly confined to platelet‐derived EVs (in the 200–900 nm size range). Accordingly, the observed sex difference reflects variation in circulating MV concentration and phenotype rather than demonstrated differences in procoagulant activity or downstream biological effect. Nevertheless, these findings suggest that platelet‐derived MV release may represent a sensitive component of sex‐specific vascular physiology under resting conditions.

In addition to the observed difference in platelet‐derived MVs, a tendency for higher CD14+/CD16‐ monocyte‐derived MVs was observed in females, although this did not reach statistical significance. This moderate effect size may suggest a biologically meaningful difference that the present study was underpowered to detect. Monocytes play a central role in innate immune activation, and prior work has demonstrated enhanced immune responsiveness in females, potentially mediated by sex hormone signaling and differential inflammatory regulation (Hoffmann et al., 2023; Klein & Flanagan, 2016). As such, elevated monocyte‐derived MV release in females may reflect sex‐specific differences in baseline immune activation or sensitivity to inflammatory stimuli. However, given the lack of statistical significance and modest sample size, this finding should be interpreted cautiously and warrants confirmation in larger, adequately powered studies.

In contrast to the selective elevation in platelet‐derived MVs, miR‐126‐3p, miR‐146a‐5p, miR‐155‐5p and miR‐181b‐5p MV expression did not differ between males and females. These miRNAs were selected based on their established roles in endothelial homeostasis, inflammatory signaling, and immune‐vascular crosstalk (Dehghani et al., 2020; Jansen et al., 2013; Prattichizzo et al., 2021). For example, miR‐126 has been linked to endothelial repair and angiogenic regulation, while miR‐146a and miR‐155 modulate Toll‐like receptor signaling and NF‐κB‐dependent inflammatory pathways (Jansen et al., 2013; Prattichizzo et al., 2021). Finally, miR‐181b has been implicated in immune cell differentiation and inflammatory responsiveness and may provide complementary insight into immune‐vascular regulation (Dehghani et al., 2020; Sun et al., 2012). The absence of sex‐related differences in MV miRNA abundance therefore suggests that, under resting physiological conditions in a healthy young cohort, biological sex may not be associated with widespread divergence in circulating MV‐linked regulatory miRNA signatures (Klein & Flanagan, 2016). Importantly, circulating miRNAs in platelet‐poor plasma are highly stable when appropriately processed, including after freeze–thaw cycles and room temperature incubation, supporting the robustness of the present measurements and reducing the likelihood that pre‐analytical factors substantially influenced the observed findings (Muth et al., 2018). However, MV uptake, target gene repression, and downstream cellular responses were not assessed. Thus, the present findings are limited to circulating MV miRNA abundance and do not address potential sex‐related differences in MV functional potency or recipient cell sensitivity.

## 5 STRENGTHS AND LIMITATIONS

This study employed standardized pre‐analytical controls and complementary analytical platforms, including SEC, nano‐flow cytometry with silica‐based reference beads, and TRPS, to quantify and phenotype MVs within a defined 200–900 nm size range. A comprehensive antibody panel and targeted MV miRNA profiling enabled simultaneous assessment of MV origin and regulatory cargo under controlled conditions. Importantly, blood sampling in females was conducted during the early follicular phase to reduce hormonal variability, and at the same time in the morning to limit diurnal variability. Finally, sex was analyzed as a primary variable rather than adjusted for as a covariate.

Several limitations warrant consideration. The study may be underpowered to detect small‐to‐moderate differences in less abundant MV subpopulations and miRNA cargo. miRNA cargo was assessed in total EV populations rather than cell‐specific EV subtypes, which may have limited the ability to detect subtype‐specific differences. Functional properties of MVs, including procoagulant activity and downstream gene regulatory effects, were not assessed; therefore, interpretation is limited to circulating MV concentration and associated miRNA abundance. Findings from this young, healthy cohort may not generalize to older or clinical populations, and as this was a secondary analysis, adjustments for multiple comparisons were not applied.

## 6 CONCLUSION

In conclusion, circulating MV profiles in healthy young adults exhibit selective sex‐related variation, characterized by higher platelet‐derived MVs and a tendency for elevated monocyte‐derived MVs in females, while total MV concentration, other MV subpopulations, and MV miRNA cargo remain comparable between sexes. These findings suggest that baseline sex differences in circulating MVs may be subtle and phenotype‐specific rather than systemic. Future studies incorporating functional assessments and broader populations are needed to determine their biological and clinical relevance.

## AUTHOR CONTRIBUTIONS

**Garett Jackson:** Data curation; formal analysis; investigation; methodology; project administration. **Hashim Islam:** Conceptualization; data curation; investigation; methodology; project administration; resources; supervision; visualization. **Alexandre Abilio de Souza Teixeira:** Data curation; formal analysis; investigation; methodology. **Christopher DeSouza:** Project administration; resources; supervision. **Isaac T. S. Li:** Project administration; resources; supervision. **Jonathan Little:** Conceptualization; funding acquisition; investigation; methodology; project administration; resources; supervision; validation.

## FUNDING INFORMATION

NSERC Discovery Grant to JPL (Grant Award Number: RGPIN‐2019‐05204). JPL was supported by a UBC Okanagan Principal's Research Chair in Metabolism (NA/0000). GJ was supported by an NSERC PGS‐D.

## CONFLICT OF INTEREST STATEMENT

The authors declare no conflict of interest.

## ETHICS STATEMENT

The study was conducted in accordance with the Declaration of Helsinki and was approved by the University of British Columbia Clinical Research Ethics Board (H20‐00240).

## Supporting information


**Figure S1.** Representative flow cytometry plots illustrating selective staining, MV distribution and silica beads for determining size boundary. (S1) 200 and 900 nm silica beads combined in one sample to provide our size range boundary of interest. (S2) Size range distribution of unstained MVs between 200 and 900 nm. (S3) Quadrant gate used to determine CD31+/CD41−, CD41+/CD31−, CD31+/CD41+ and CD31−/CD41− populations based on an FMO protocol gating strategy.

## Data Availability

De‐identified data that support the findings of this study are available from the corresponding author upon reasonable request, subject to institutional ethics and privacy requirements.
