# Supplementary material for: Sex differences in circulating platelet‐derived CD41+ extracellular vesicles in healthy adults
Source: Physiol Rep. 2026 Jul 3;14(13):e70932. doi: 10.14814/phy2.70932 (PMC13332322; doi:10.14814/phy2.70932)
Supplement: Supplementary file 1 — Figure S1. Representative flow cytometry plots illustrating selective staining, MV distribution and silica beads for determining size boundary. (S1) 200 and 900 nm silica beads combined in one sample to provide our size range boundary of interest. (S2) Size range distribution of unstained MVs between 200 and 900 nm. (S3) Quadrant gate used to determine CD31+/CD41−, CD41+/CD31−, CD31+/CD41+ and CD31−/CD41− populations based on an FMO protocol gating strategy. [file PHY2-14-e70932-s001.docx]

**
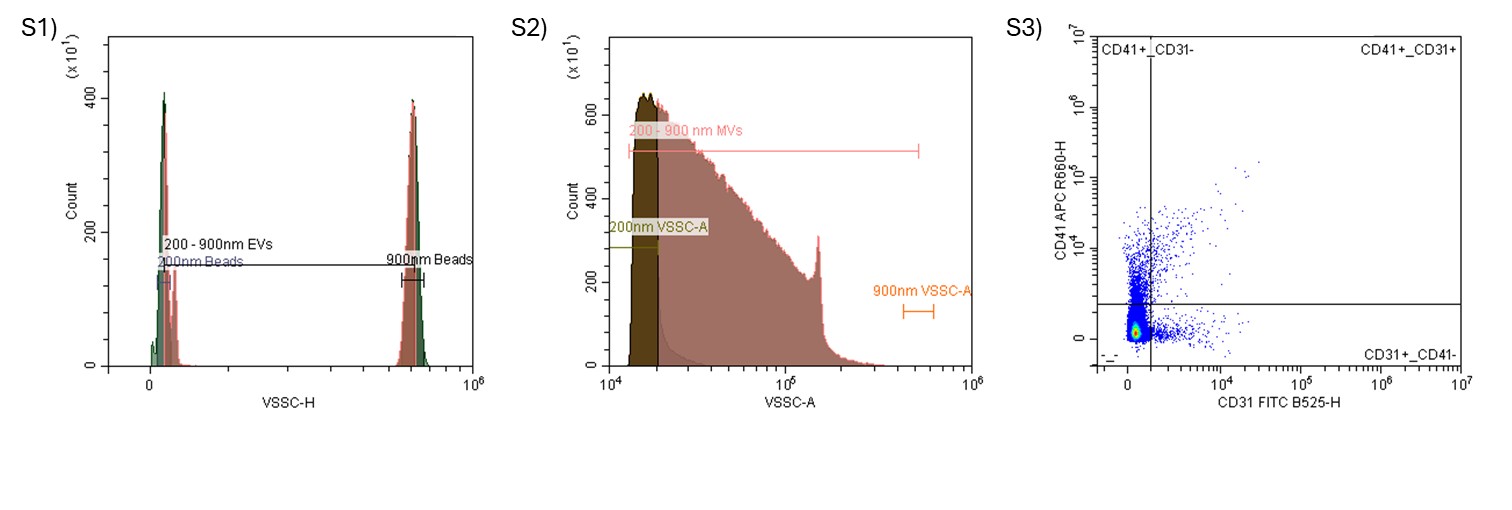
**

**Supplementary Figure 1.** Representative flow cytometry plots illustrating selective staining, MV distribution and silica beads for determining size boundary. S1) 200 and 900 nm silica beads combined in one sample to provide our size range boundary of interest. S2) Size range distribution of unstained MVs between 200 and 900 nm. S3) Quadrant gate used to determine CD31+/CD41-, CD41+/CD31-, CD31+/CD41+ and CD31-/CD41- populations based on an FMO protocol gating strategy.
